# Susceptibility to Oxidative Stress Is Determined by Genetic Background in Neuronal Cell Cultures

**DOI:** 10.1523/ENEURO.0335-17.2018

**Published:** 2018-03-19

**Authors:** Mattias Günther, Faiez Al Nimer, Fredrik Piehl, Mårten Risling, Tiit Mathiesen

**Affiliations:** 1 Department of Neuroscience, Karolinska Institutet, Stockholm, Sweden; 2 Department of Clinical Neuroscience, Karolinska Institutet, Stockholm, SE-171 77, Sweden

**Keywords:** oxidative stress, neuronal inflammation, traumatic brain injury, cell culture, Dark Agouti, Piebald Viral Glaxo

## Abstract

Traumatic brain injury (TBI) leads to a deleterious and multifactorial secondary inflammatory response in the brain. Oxidative stress from the inflammation likely contributes to the brain damage although it is unclear to which extent. A largely unexplored approach is to consider phenotypic regulation of oxidative stress levels. Genetic polymorphism influences inflammation in the central nervous system and it is possible that the antioxidative response differs between phenotypes and affects the severity of the secondary injury. We therefore compared the antioxidative response in inbred rat strains dark agouti (DA) to piebald viral glaxo (PVG). DA has high susceptibility to inflammatory challenges and PVG is protected. Primary neuronal cell cultures were exposed to peroxynitrite (ONOO^−^), nitric oxide (NO), superoxide (O_2_^−^), and 4-hydroxynonenal (4-HNE). Our findings demonstrated a phenotypic control of the neuronal antioxidative response, specific to manganese O_2_^−^ dismutase (MnSOD). DA neurons had increased levels of MnSOD, equal levels of peroxiredoxin 5 (PRDX5), decreased oxidative stress markers 3-nitrotyrosine (3-NT) and 4-HNE and decreased neuronal death detected by lactate dehydrogenase (LDH) release after 24 h, and higher oxidative stress levels by CellROX than PVG after 2 h. It is possible that DA neurons had a phenotypic adaptation to a fiercer inflammatory environment. ONOO^−^ was confirmed as the most powerful oxidative damage mediator, while 4-HNE caused few oxidative effects. Inducible NO synthase (iNOS) was not induced, suggesting that inflammatory, while not oxidative stimulation was required. These findings indicate that phenotypic antioxidative regulation affects the secondary inflammation, which should be considered in future individualized treatments and when evaluating antioxidative pharmacological interventions.

## Significance Statement

Neurotrauma leads to inflammation and oxidative stress in the brain. The outcome differs between individuals, and it is largely unknown what causes this diversity. It is possible that the brain phenotype is linked to oxidative stress levels, and that some individuals acquire less oxidative stress than others. We therefore tested the oxidative stress reaction patterns in rat neurons from two strains with different susceptibility to inflammation. We found that the phenotypes have different regulation of antioxidative enzymes and oxidative stress. While further studies are needed to corroborate the findings *in vivo*, it is a proof of concept of genetic regulation of direct oxidative stress, which may impact outcome after TBI and interact with future antioxidative treatment trials.

## Introduction

Traumatic brain injury (TBI) leads to a multifactorial and mostly deleterious secondary inflammatory response in the brain. The degree of injury is related to the severity of the inflammation. Directly after the primary trauma, extravasation of neutrophils, blood-brain barrier damage, astrocyte and microglia activation, migration of leucocytes and phagocytes and cytokine and chemokine production occurs (Morganti-Kossmann et al., 2007). These events create oxidative stress. Reactive oxygen species (ROS) and reactive nitrogen species (RNS) overwhelm the antioxidative response, react with proteins, lipids, carbohydrates and nucleic acids, which results in irreversible cellular damage (Bains and Hall, 2011).

Outcome in TBI varies considerably. The difference of individual responses to trauma is considered a major cause to why experimental head injury findings are difficult to apply to clinical trauma and to why trials in neuroprotection for human TBIs have failed (Maas and Menon, 2012). It is possible that individual differences in the antioxidative defense affect the severity of the secondary injury, and it was hypothesized that genetic host factors, such as individual inflammatory responses to traumatic stimuli that were defined for DA and PVG rats (Al Nimer et al., 2013) would be one explanatory factor for heterogeneous outcomes. Unexpectedly, large differences in inflammatory responses did not correlate with discernible differences in posttraumatic neuronal death (Günther et al., 2012). The animals seemed robustly armed to deal with the inflammatory challenge despite inter-strain differences in inducible nitric oxide synthase (iNOS) production, which had been hypothesized to correlate with neuronal death; each animal seemed to respond appropriately on a system level. Recently, immunologic responses have been studied on a system level (Aderem and Smith, 2004; Brodin and Davis, 2017) and a system level explanation would fit the fact that not only the potentially damaging iNOS was upregulated in one strain, but also manganese O_2_^−^ dismutase (MnSOD), that would protect by decreasing substrates for peroxynitrite (ONOO^−^) formation. Genetic polymorphisms influence the inflammatory activity in the central nervous system (Jordan, 2007; McAllister, 2010; Dardiotis et al., 2010), but it is unknown to what extent this affects oxidative stress in traumatic injury. Transgenic animals have been manipulated to study the impact of single genes on oxidative stress (Misawa et al., 2006; Holley et al., 2011); mutations that affected MnSOD were either lethal or seemed to correlate with adaptive reactions. The genetic similarity in inbred animals is due to preserved spontaneous mutations, which is why inbred animals comprise a biological system rather than a single genetic abnormality. Inbred animals offer models to study differences in inflammatory responses between genetically similar groups of animals on a system level and are in that aspect more similar to the clinical situation. A patient represents a biological system with its unique and spontaneous genetic make-up. We therefore compared the neuronal antioxidative response in inbred rat strains dark agouti (DA) and piebald viral glaxo (PVG). DA has high susceptibility to, and PVG is protected from CNS inflammation connected to TBI, experimental autoimmune encephalomyelitis, nerve axotomy and spinal cord injury (Reid et al., 2010; Al Nimer et al., 2011). DA responds with increased levels of macrophages, granulocytes, NK-cells, microglia and complement factors C3, C1q, and CD11b compared to PVG after TBI (Bellander et al., 2010; Günther et al., 2012; Al Nimer et al., 2013). Inflammatory cells induce ROS in the CNS (Block et al., 2007). C1q-/- mice neurons had lower oxidative stress after hypoxia/ischemia (Ten et al., 2010). C3-/- mice had better outcome after brain ischemia (Mocco et al., 2006). We hypothesized that the phenotypes of DA and PVG would differ in the regulation of the antioxidative response, oxidative stress levels and ultimately cell survival. The aim was to determine whether these genetically unique strains would respond according to individual patterns when subjected to oxidative challenges *in vitro* and whether such patterns could be determined and described. The cell culture environment is void of inflammatory cells and circulating cytokines which allows for the identification of an inherent neural antioxidative response.

**Figure 1.**
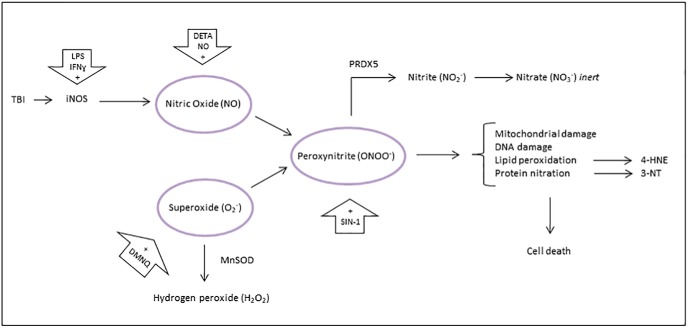
Primary neuronal cell cultures were exposed to key oxidants in TBI: ONOO^−^, NO, and O_2_^−^. NO reacts with O_2_^−^ to form ONOO^−^. ONOO^−^ causes protein nitration, lipid peroxidation, DNA damage, and inhibition of mitochondrial electron transport, leading to necrotic cell death. O_2_^−^ is removed by MnSOD and ONOO^−^ is removed by PRDX5.

Primary neuronal cell cultures were exposed to key oxidants in TBI; NO, superoxide (O_2_^−^), and ONOO^−^ (Bains and Hall, 2011; Fig. 1). NO reacts with O_2_^−^ to form ONOO^−^ (Faraci, 2006; Lambert and Brand, 2009). ONOO^−^ causes protein nitration, lipid peroxidation, DNA damage and inhibition of mitochondrial electron transport, leading to necrotic cell death (Lu et al., 2009). Isolated neurons were selected due to their particular vulnerability to oxidative stress. Postmitotic neurons cannot divide to replace or dilute damaged components, and have low levels of antioxidants compared to glia (Almeida et al., 2002).

Antioxidative enzymes MnSOD and peroxiredoxin 5 (PRDX5) were compared and correlated to markers of lipid peroxidation; 4-hydroxynonenal (4-HNE), protein nitration; 3-nitrotyrosine (3-NT) and neuronal death detected by lactate dehydrogenase (LDH) release. In addition, neuronal iNOS induction was investigated together with direct oxidative effects of 4-HNE.

## Materials and Methods

### Primary neuronal cultures

All animal procedures were performed in accordance with the Karolinska Institutet Animal Care Committee’s regulations. The DA strain was originally obtained from Medizinische Hochschule, Hannover, Germany while the PVG.AV1 strain was obtained from Harlan UK Ltd. All animals were bred in an in-house breeding facility with 12/12 h light/dark cycles and fed standard rodent chow and water *ad libitum*. Four female DA and PVG rats were simultaneously pared with respective males for 72 h. Pregnant rats were asphyxiated by CO_2_ 18 days later, ensuring an embryonic post gestation age between E18 and E21. Hippocampal neuronal cultures from DA and PVG were prepared simultaneously by dissecting the embryonic hippocampi before dissociation by trypsin (Life Technologies) in 37°C for 15 min followed by mechanical dissociation by a Pasteur pipette. The cell concentration was determined in the suspension by Countess automated cell counter (Life Technologies) and cells were seeded at 3 × 10^5^ cells/well and placed in Nunclon 24- or 48-well plates (Thermo Scientific), coated with poly-L-lysine (Sigma-Aldrich). The cells were kept in Neurobasal medium supplemented with B_27_, 200 mM L-glutamine, and 15 µg/ml gentamicin (Life Technologies). The B_27_ supplement contained antioxidants vitamin E, vitamin E acetate, SOD, catalase, and glutathione. The neuronal-glial ratio was >98% determined by immunofluorescent double staining with NeuN and GFAP (data not shown). No differences were seen in fetus count per pregnancy, fetal size, cell count at seeding and average cell size at seeding, ensuring equal conditions at oxidative provocation (data not shown).

### Oxidative stress

Twenty-four hours after seeding, the medium was changed to Neurobasal medium with B_27_ void of antioxidants. The cells were exposed to oxidative stress for 2–24 h. For the oxidative stress analysis at 2 h, parallel cultures were prepared with B_27_ containing antioxidants, to determine reversibility. Oxidative stress was produced by the following compounds. (1) Diethylenetriamine/NO adduct (DETA NO) releases 2 M NO/mol parent compound (Sigma Aldrich). A stock was prepared (50 mM) in dH_2_O, which was diluted in cell culture medium in concentrations according to previous studies (Dranka et al., 2010, 2011). (2) 2,3-Dimethoxy-1,4-naphthoquinone (DMNQ) releases O_2_^−^ (Sigma Aldrich). A stock was prepared (15 mM) in DMSO, which was diluted in cell culture medium in concentrations according to previous studies (Tamm et al., 2008; Dranka et al., 2010, 2011). The concentration of DMSO in cell culture medium did not exceed 0.1%. (3) 3-Morpholinosydnonimine hydrochloride (SIN-1) uses molecular oxygen to generate both O_2_^−^ and NO that spontaneously form ONOO^−^ (Sigma Aldrich). A stock was prepared (3 mM) in dH_2_O, which was diluted in cell culture medium to concentrations according to previous studies (Trackey et al., 2001; Acquaviva et al., 2004). (4) 4-HNE is formed by peroxidation of fatty acids (Calbiochem). The stock was supplied at 10 mg/ml and diluted in cell culture medium to concentrations according to previous studies (Malecki et al., 2000; Dranka et al., 2011). Physiologic cellular concentrations are in the range of 0.1 to 3.0 μM but may increase to 10 μM to 5 mM by oxidative stress (Dianzani, 2003).

### Western blotting

After 24 h of oxidative stress, cells were washed with 4°C HBSS. RIPA lysis buffer (TBS, 1% Nonidet P-40, 0.5% sodium deoxycholate, 0.1% SDS, 0.004% sodium azide, PMSF, protease inhibitor cocktail, and sodium orthovanadate) was added for 15 min at 4°C (Santa Cruz Biotechnology). Cells were scraped from the bottom of the wells and placed in plastic tubes (six to eight wells were combined in one sample) and centrifuged for 10 min at 10,000 rpm at 4°C. The protein content was determined in the supernatant by a protein assay (Bio-Rad). Samples were denaturated (70°C, 10 min) and reduced (2.5% β-mercaptomethanol), and loaded on NuPAGE Novex Bis-Tris 10% mini gels (Life Technologies) with Odyssey protein molecular weight marker (Li-Cor). Electrophoresis and transfer to PVDF membranes were done in XCell SureLock Mini-Cell, with buffers according to manufacturer’s instructions (Life Technologies). Membranes were blocked for 1h in Odyssey blocking buffer (Li-Cor) and incubated overnight in 4°C with primary antibody and α-tubulin loading control diluted in Odyssey blocking buffer. Membranes were washed 4× 5 min in PBS + 0.1% Tween 20 and incubated in secondary antibodies diluted in Odyssey blocking buffer for 1h, followed by washing 5× 5 min in PBS + 0.1% Tween 20 before being scanned by Odyssey infrared imaging system (Li-Cor), allowing two antibodies to be detected simultaneously in 700 and 800 nm. Densiometric quantification and normalization to α-tubulin were done in Image Studio v.2.1 (Li-Cor). All membranes contained an identical sample from rat macrophage cell line NR8383, stimulated with 500 ng/ml lipopolysaccharide (LPS) from E-coli 0128:B12 (Sigma-Aldrich) and 100 ng/ml recombinant rat interferon gamma (IFN-ɣ) (Millipore) for 24 h. The NR8383 sample expressed all proteins/protein-adducts examined allowing all membranes to be normalized to the sample, removing natural differences in Western blotting processing and staining and allowing for comparisons between the membranes. A total of 77 membranes were quantified and normalized to the NR8383 control. Primary and secondary antibodies are specified in Table 1. Protein-HNE adducts and 3-NT were quantified at 36/42 kDa.

**Table 1.** List of antibodies and reagents used

Antibody/assay	Specificity	Species	Source	Product number	Dilution
MnSOD	SOD2	Rb	Abcam	ab13533	1:5000
PRDX5	Peroxyredoxin V	Rb	Abcam	ab180587	1:1000
4-HNE	4 Hydroxynonenal	Rb	Abcam	ab46545	1:1000
3-NT	3-Nitrotyrosine [39B6]	Mo	Abcam	ab61392	1:500
iNOS	Inducible NO synthase	Rb	Abcam	ab15323	1:250
α-Tubulin	Loading control	Mo	Abcam	ab7291	1:10,000
α-Tubulin	Loading control	Rb	Abcam	ab176560	1:1000
NeuN	Neuron-specific nuclear protein	Mo	Millipore	MAB377	1:500
GFAP	Glial fibrillary acidic protein	Rb	Abcam	ab33922	1:1000
Alexa Fluor 680	Secondary 680 nm	GoαRb	Molecular Probes	A21076	1:15,000
800CW	Secondary 800 nm	DoαMo	Li-Cor	926-32212	1:15,000
800CW	Secondary 800 nm	GoαRb	Li-Cor	926-32211	1:15,000
LDH assay	Lactate dehydrogenase release	N/A	Abcam	ab102526	N/A

### LDH assay

LDH is an oxidoreductase present in all cell types. LDH is released into cell culture medium relative to the loss of cell membrane integrity, thus a marker of necrotic cell damage. LDH activity in cell culture medium was measured by a colorimetric assay (Abcam). LDH reduces NAD to NADH, which interacts with a specific probe to produce a color (λmax = 450 nm), quantified by Multiskan EX plate reader (Thermo Fisher Scientific). A standard curve was constructed and the LDH activity was measured and calculated according to the manufacturer instructions and found to be 5.81–24.05 nmol/min/ml = mU/ml, which was within the range of the assay (1–100 mU/ml). The LDH activity in the medium was normalized to the total protein amount in the corresponding wells, quantified for Western blotting as previously described.

### Cell-IQ

Cells were photographed at 0 and 24 h by Cell-IQ live cell imaging and analysis platform (Chipman Technology), a 10× phase contrast microscope in an incubator setting.

### CellROX oxidative stress detection

CellROX green reagent is a fluorogenic probe for measuring oxidative stress in live cells. The cell-permeant dye is weakly fluorescent while in a reduced state but exhibits bright green photostable fluorescence on oxidation by ROS and subsequent binding to DNA, with absorption/emission maxima of ∼485/520 nm (GFP; Life Technologies). CellROX was added to the wells in a 5 µM final concentration after 2 h of oxidative stress. NucBlue reagent, a Hoechst 33342 cell-permeant nuclear counterstain, was added for 15 min (Life Technologies). After 30 min, the cells were washed two times with 4°C HBSS. The cell culture plates were photographed in 20× magnification in a Zeiss Observer Z-inverted microscope. For each view, a DAPI and a GFP picture were quantified in CellProfiler (Jones et al., 2008) by measuring the integrated intensity of the GFP staining at the loci of corresponding DAPI staining, thus measuring oxidative stress level per cell.

### Statistical analyses

Statistical analyses were done by GraphPad Prism version 6.05 for Windows (GraphPad Software). All results were related to the baseline of that particular assay, probe and strain, and presented as percentage of the baseline, allowing for comparisons between experiments; α-level *p* < 0.05 was considered significant. All error bars represent the standard error of the mean. CellROX, Western blottings, and LDH assays were analyzed by two-way ANOVAs with Šídák´s multiple comparisons test. Baselines were analyzed by the nonparametric Mann–Whitney test.

## Results

### MnSOD

**Figure 2.**
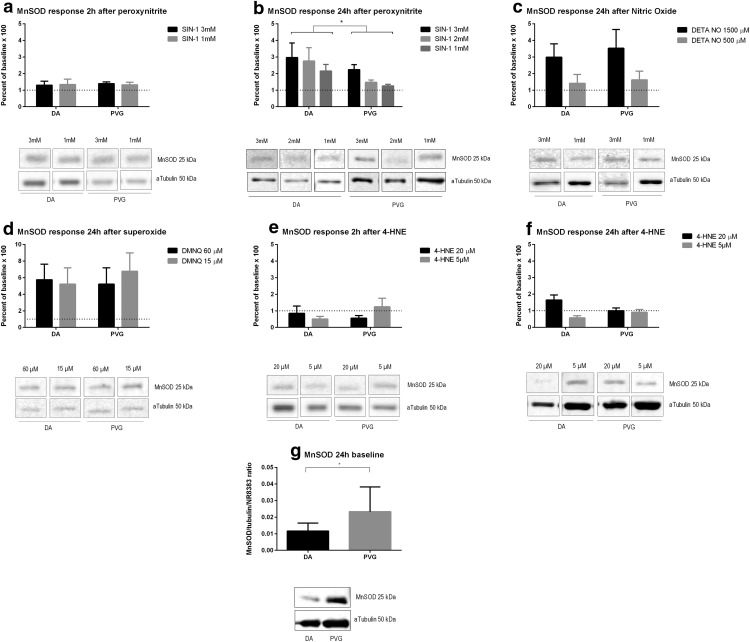
Genetic background regulated antioxidative enzyme MnSOD after oxidative stress. ONOO^−^ caused higher MnSOD synthesis in DA compared to PVG. Baseline levels were higher in PVG compared to DA. No increase was seen at 2 h, confirming a *de novo* protein synthesis. Dotted lines mark baselines. Pictures are constructed from different parts of gels and marked as such. Densiometric quantification was made as a mean of three consecutive gels which were normalized to both α-tubulin and a specific control identical for all gels; *p < 0.05.

ONOO^−^ did not induce MnSOD compared to controls at 2 h (Fig. 2*A*). At 24 h, MnSOD was induced in DA compared to PVG (*p* < 0.05; Fig. 2*B*). NO induced MnSOD at 24 h equally in DA and PVG (Fig. 2*C*). O_2_^−^ induced MnSOD at 24 h equally in DA and PVG (Fig. 2*D*). 4-HNE did not induce MnSOD compared to controls at 2 or 24 h (Fig. 2*E*,*F*). Baseline MnSOD levels were higher in PVG compared to DA (*p* < 0.05; Fig. 2*G*).

### PRDX5

**Figure 3.**
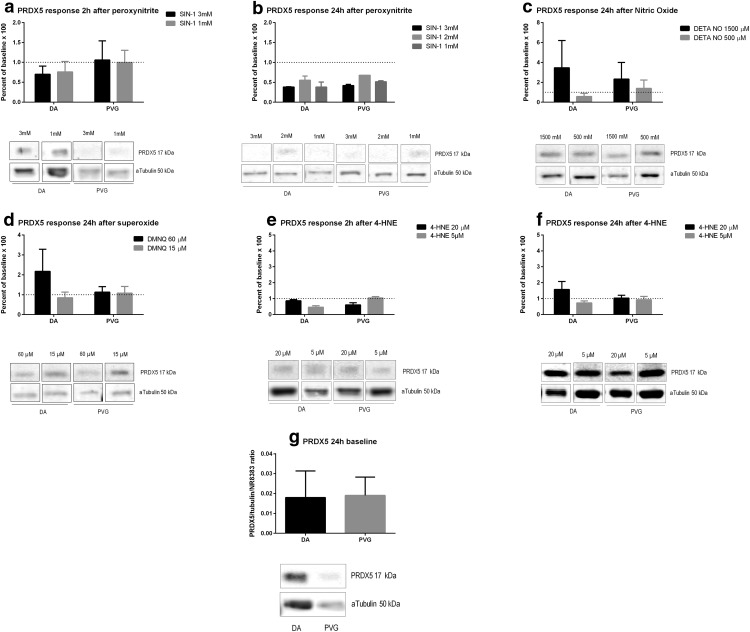
Genetic background did not regulate antioxidative enzyme PRDX5 after oxidative stress. PRDX5 was decreased by ONOO^−^, increased by NO and O_2_^−^, and unchanged by 4-HNE. Baseline expression did not differ between strains. Dotted lines mark baselines. Pictures are constructed from different parts of gels and marked as such. Densiometric quantification was made as a mean of three consecutive gels, which were normalized to both α-tubulin and a specific control identical for all gels.

ONOO^−^ reduced PRDX5 at 2 h (Fig. 3*A*) and further at 24 h (Fig. 3*B*), equally in DA and PVG. NO induced PRDX5 at 24 h equally in DA and PVG (Fig. 3*C*). O_2_^−^ induced PRDX5 only at 60 µM at 24 h, equally in DA and PVG (Fig. 3*D*). 4-HNE did not induce PRDX5 in either DA or PVG at 2 h (Fig. 3*E*) or 24 h (Fig. 3*F*). Baseline PRDX5 expression did not differ between DA and PVG (Fig. 3*G*).

### 4-HNE

**Figure 4.**
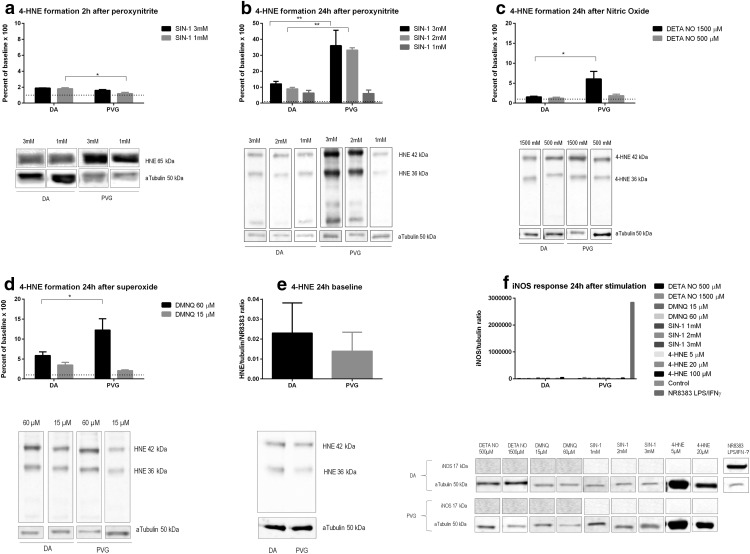
Genetic background effected 4-HNE formation after oxidative stress. PVG had higher levels of 4-HNE after oxidative stress by ONOO^−^, NO, and O_2_^−^ compared to DA at 24 h. A discrete increase was seen at 2 h. ONOO^−^ caused a 35× increase of 4-HNE in PVG, compared to 7× by NO and 12× by O_2_^−^, why ONOO^−^ was confirmed as the most powerful oxidant. Baseline levels did not differ between strains. Neuronal iNOS was not induced by any of the oxidants ONOO^−^, NO, O_2_^−^, or 4-HNE. Dotted lines mark baselines. Pictures are constructed from different parts of gels and marked as such. Densiometric quantification was made as a mean of three consecutive gels which were normalized to both α-tubulin and a specific control identical for all gels; *p < 0.05 and **p < 0.01.

ONOO^−^ increased 4-HNE at 2 h, with a higher increase in DA compared to PVG at 1 mM (*p* < 0.05; Fig. 4*A*). At 24 h, 4-HNE formation was instead significantly increased in PVG compared to DA at 2 mM (*p* < 0.01) and 3 mM (*p* < 0.01; Fig. 4*B*). NO increased 4-HNE at 24 h in PVG compared to DA (*p* < 0.05; Fig. 4*C*). O_2_^−^ increased 4-HNE at 24 h in PVG compared to DA (*p* < 0.05; Fig. 4*D*). Baseline 4-HNE did not differ between DA and PVG Fig. 4*E*).

### 3-NT

**Figure 5.**
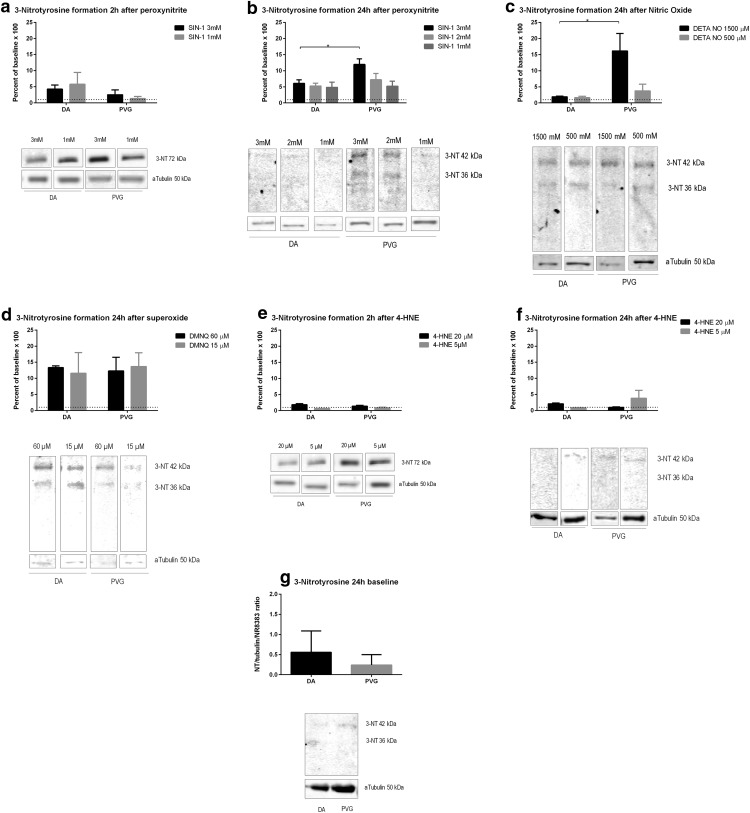
Genetic background effected 3-NT formation after oxidative stress. PVG had higher levels of 3-NT after oxidative stress by ONOO^−^ and NO. ONOO^−^, NO, and O_2_^−^ consistently caused a 10–15× increase of 3-NT, why nitrosylation occurred indiscriminate of oxidant. A discrete increase was seen at 2 h. Baseline levels did not differ between strains. Dotted lines mark baselines. Pictures are constructed from different parts of gels and marked as such. Densiometric quantification was made as a mean of three consecutive gels which were normalized to both α-tubulin and a specific control identical for all gels; *p < 0.05.

ONOO^−^ increased 3-NT at 2 h equally in DA and PVG (Fig. 5*A*), and at 24 h, 3-NT increased in PVG compared to DA at 3 mM (*p* < 0.05; Fig. 5*B*). NO increased 3-NT at 24 h higher in PVG compared to DA at 1500 µM (*p* < 0.05; Fig. 5*C*). O_2_^−^ increased nitrotyrosine at 24 h equally in DA and PVG (Fig. 5*D*). 4-HNE did not increase 3-NT in either DA or PVG at 2 or 24 h (Fig. 5*E*,*F*). Baseline nitrotyrosine did not differ between DA and PVG (Fig. 5*G*).

### Acute neuronal oxidative stress detected by CellROX

**Figure 6.**
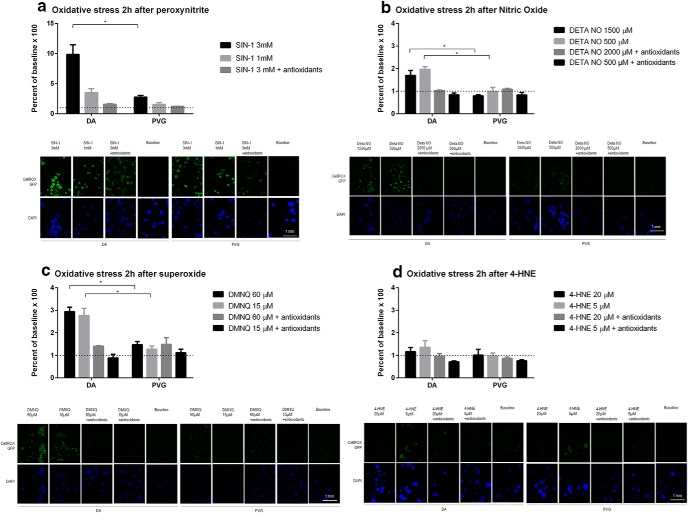
Genetic background effected the acute oxidative stress at 2 h. Oxidative stress levels measured by CellROX fluorescent marker were consistently higher in DA after NO, O_2_^−^, and ONOO^−^, compared to PVG. ONOO^−^ caused a 10× increase of oxidative stress compared to a 2× increase for NO and 3× for O_2_^−^ why ONOO^−^ was confirmed as the most powerful oxidant. 4-HNE did not cause oxidative stress in the neurons. Dotted lines mark baselines; *p < 0.05.

ONOO^−^ resulted in dose dependent oxidative stress at 2 h, with higher levels in DA compared to PVG at 3 mM (*p* < 0.05; Fig. 6*A*). This effect was fully reversed by the addition of antioxidants. NO resulted in oxidative stress at 2 h, with higher levels in DA compared to PVG at 1500 µM (*p* < 0.05) and 500 µM (*p* < 0.05; Fig. 6*B*). This effect was fully reversed by the addition of antioxidants. O_2_^−^ resulted in oxidative stress at 2 h, with higher levels in DA compared to PVG at 15 µM (*p* < 0.05) and 60 µM (*p* < 0.05; Fig. 6*C*). This effect was fully reversed by the addition of antioxidants. 4-HNE did not cause oxidative stress at 2 h in either DA or PVG (Fig. 6*D*).

### Cell death detected by LDH release

**Figure 7.**
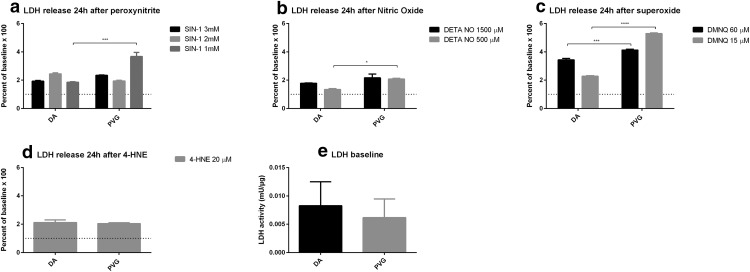
Genetic background effected neuronal death, measured by LDH, after oxidative stress. PVG had increased neuronal death after oxidative stress consistently by ONOO^−^, NO, and O_2_^−^ compared to DA at 24 h. Baseline levels did not differ between strains. Dotted lines mark baselines; *p < 0.05, ***p < 0.005, and ****p < 0.001.

ONOO^−^ caused increased cell death in PVG compared to DA at 1mM (*p* < 0.005; Fig. 7*A*). NO caused increased cell death in PVG compared to DA at 500 µM (*p* < 0.01; Fig. 7*B*). O_2_^−^ caused increased cell death in PVG compared to DA at 15 µM (*p* < 0.001) and 60 µM (*p* < 0.001; Fig. 7*C*). 4-HNE caused equal levels of cell death in DA and PVG (Fig. 7*D*). Baselines of LDH were equal between DA and PVG (Fig. 7*E*).

### iNOS

iNOS was not induced by any of the oxidants ONOO^−^, NO, O_2_^−^, or 4-HNE (Fig. 4*F*).

### Cell-IQ

**Figure 8.**
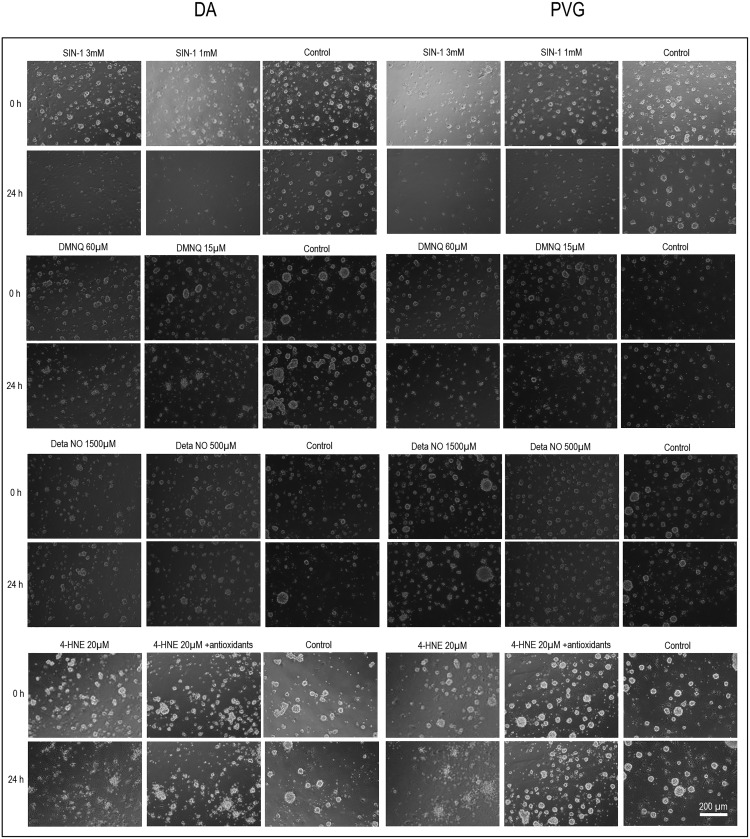
Photomicrographs by Cell-IQ of neuronal cell cultures. Morphologic changes were detected after ONOO^−^ (SIN-1), NO (DETA NO), O_2_^−^ (DMNQ), and 4-HNE. Control cultures did not exhibit signs of cell death. Cell death was extensive after ONOO^−^ compared to NO, O_2_^−^, and 4-HNE. Morphologic signs of cell death were reversed in the 4-HNE groups by the addition of antioxidants.

Morphologic changes in the neurospheres were detected consistently after ONOO^−^, NO, O_2_^−^, and 4-HNE provocation at 24 h. Baseline cultures did not exhibit signs of cell death. Cell death was extensive after ONOO^−^ compared to NO, O_2_^−^, and 4-HNE. Differences in cell death based on morphology between DA and PVG could not be established. Morphologic signs of cell death were reversed in the 4-HNE groups by the addition of antioxidants (Fig. 8).

## Discussion

**Figure 9.**
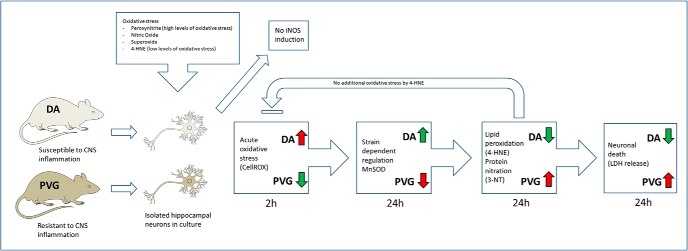
Graphic presentation of the phenotype regulated neuronal antioxidative response.

The findings of this study suggest genetically encoded biologically relevant differences in the response of the neuron to oxidative stress (Fig. 9). Neurons from DA and PVG rats appeared to represent two different response patterns to oxidative challenges. Each mode entailed a robust and balanced reaction which may minimize cellular injury and could be viewed as system on a cellular level. In particular, variability in the regulation of MnSOD may explain some of the strain differences, where MnSOD was increased to a greater extent in DA neurons, likely due to a *de novo* protein synthesis since no increase was detected early. MnSOD converts O_2_^−^ to hydrogen peroxide (H_2_O_2_) in mitochondria which limits oxidative stress in TBI (Flynn and Melov, 2013). O_2_^−^ caused a 6× MnSOD induction compared to 3× by NO and 1.5× by ONOO^−^, confirming O_2_^−^ as a main trigger for MnSOD induction. The phenotype difference was observed specifically by ONOO^−^ but not O_2_^−^ or NO. It is possible that greater initial oxidative stress was required for effective MnSOD induction in DA. This notion is supported by the fact that ONOO^−^ caused the highest oxidative stress levels (10×) compared to NO (2×) and O_2_^−^ (3×), and extensive morphologic signs of cell death in Cell-IQ. Interestingly, our findings are to some degree unexpected, since the DA strain has been associated with a higher degree of inflammation and oxidative damage in *in vivo* models for nerve trauma (Piehl et al., 1999; Lundberg et al., 2001). It can therefore be speculated if the pro-inflammatory phenotype of DA has led to higher resilience to oxidative stress in nerve cells, thus an example of hormesis. In other models both repeated oxidative stress by H_2_O_2_ and ischemic precondition led to adaptive and increased protective mechanisms in cell cultures (Pickering et al., 2013; Kalogeris et al., 2014). Further studies are needed to clarify the impact of long-term genetic adaptation to oxidative stress and the mechanisms thereof. In contrast, PRDX5 induction was equal in DA and PVG, consistently for all oxidants. PRDX5 is a selective ONOO^−^ reductase which protects from oxidative stress in TBI (Szabó et al., 2007). NO and O_2_^−^ caused increased PRDX5 synthesis and ONOO^−^ caused depletion. The phenotype regulated antioxidative response was therefore not a result of a general increase of antioxidative systems, but specific for MnSOD. Lipid peroxidation marker 4-HNE was consistently decreased in DA compared to PVG after NO, O_2_^−^, and ONOO^−^. 4-HNE is an α,β-unsaturated aldehyde generated by peroxidation of ω-6 polyunsaturated fatty acids. The initial oxidative stress and subsequent lipid peroxidation correlated, since ONOO^−^ resulted in a 35× increase of 4-HNE in PVG, compared to 7× by NO and 12× by O_2_^−^. It may be that higher MnSOD levels in DA provided a higher degree of protection from lipid peroxidation. At physiologic concentrations, 4-HNE acts as an endogenous signaling molecule but causes neuronal death in high concentrations (Kruman and Mattson, 1999; Uchida, 2003). It is possible that neurons respond specifically to pathologic 4-HNE concentrations by increasing antioxidative enzymes similarly to the response to NO, O_2_^−^, and peroxyntrite. 4-HNE induces peroxyredoxins in macrophages (Ishii et al., 2004). Direct exposure of the neurons to exogenous 4-HNE was therefore investigated, which failed to induce MnSOD or PRDX5 in either strain. No oxidative stress was detected at 2 h, and protein nitration marker 3-NT was not elevated. The neurons seemed relatively resistant and unresponsive to toxic effects by 4-HNE. Cell death measured by LDH release was half compared to NO, O_2_^−^, and ONOO^−^ and no morphologic signs of cell death were detected. It is probable that toxicity associated with 4-HNE in the whole brain is an effect of the general oxidative stress environment. Neurons may therefore lack specific endogenous defense systems to 4-HNE and be dependent on an antioxidative response by surrounding cells such as glia cells. Protein nitration marker 3-NT was lower in DA compared to PVG after ONOO^−^ and NO. O_2_^−^ caused no difference in 3-NT levels between phenotypes. 3-NT is caused by nitrosylation of tyrosine residues (Beckman et al., 1990) and is found in cortical tissue in TBI (Deng et al., 2007). 3-NT was consistently increased 10–15× in PVG neurons after NO, O_2_^−^, and ONOO^−^. Nitrosylation therefore occurred indiscriminate of oxidant and correlated with the initial oxidative stress levels and MnSOD expression, similarly to lipid peroxidation. MnSOD and PRDX5 effectively eliminate O_2_^−^ and ONOO^−^, but no specific enzyme targets NO. NO in low concentrations controls diverse physiologic functions such as cytotoxicity, cytostasis, regulation of vascular tone, inhibition of platelet aggregation and neurotransmission. In higher concentrations NO becomes toxic by reacting with O_2_^−^ to form ONOO^−^ (O'Connell and Littleton-Kearney, 2013). A majority of inflammatory derived NO is caused by iNOS, mostly in inflammatory cells but also in neurons (Günther et al., 2012). It is possible that oxidative stress leads to direct neuronal iNOS induction, which would add to the oxidative insult and the neurotoxicity. iNOS expression was therefore investigated and was not induced by ONOO^−^, NO, O_2_^−^, or 4-HNE in either DA or PVG. It is likely that *de novo* synthesis required inflammatory networks and that isolated oxidative stress alone was not sufficient for neuronal iNOS induction.

Factors limiting the extrapolation of results to the *in vivo* situation includes the use of higher O_2_ and CO_2_ tensions in cell cultures compared to *in vivo*, which may influence how cells respond to oxidative stress. Moreover, the study involved a single cell type which may react differently *in vivo*, in a context with surrounding cells in the brain such as glia, endothelium and immune cells. Nevertheless, a reductionist approach was necessary to test the hypothesis of phenotypic differences in nerve cells specifically. Neurons from both strains were treated identically and reacted differently why results should be regarded as proof of concept while not directly transferable to how neurons perform *in vivo*. Earlier studies of knock-out animals have established that manipulating specific enzymes may affect the redox balance (Holley et al., 2011). We have used an alternative approach to test genetic control of oxidative insults. Our study presented novel findings of genetic control of redox processes, in inbred and not engineered genomes. Knock-out models may be difficult to interpret and do not represent spontaneously generated systems; conditional knock-out of MnSOD in postnatal neurons in mice did not increase oxidative damage (Misawa et al., 2006). Furthermore, our study provides a systems approach to the inflammatory challenge and its potential to cause neuronal death. The experimental rats responded with higher iNOS and MnSOD levels in the PVG strain (Günther et al., 2012) while isolated neuronal cultures in this study showed a different response when the systems were reduced to individual cells: now DA rats responded with early inflammatory markers and higher MnSOD-levels, while PVG rats were actually more susceptible to the challenges, produced more 3-NT and produced higher levels of LDH as markers of cellular death. Taken together, the findings provided a model to view individuals as integrated biological systems with sets of balanced responses to individual inflammatory stimuli; a systems view that is used for studies of the immune system (Aderem and Smith, 2004; Brodin and Davis, 2017). In this context, studies of inflammatory challenges may integrate the span from reduced single biochemical reactions to cellular reactions and intercellular interplay that form a biological totality that may better explain heterogonous and seemingly contradictory responses to trauma and treatment.

## Conclusion

The neuronal antioxidative response and oxidative stress levels varied with the oxidant used, where ONOO^−^ was confirmed as the most powerful mediator of oxidative damage, while 4-HNE caused mild effects. 4-HNE and iNOS did not cause additional oxidative damage. DA neurons displayed a higher antioxidative response, resulting in lower oxidative stress and cell death mainly due to a stronger induction of MnSOD and not a general increase of antioxidative systems. It can be speculated if this represents an adaption of DA neurons to a more inflammation-prone environment. These findings indicate that phenotypic antioxidative regulation affects the secondary inflammation and oxidative stress in TBI, which should be considered in future individualized treatments and when evaluating antioxidative pharmacological interventions.

## Synthesis

Reviewing Editor: Maiken Nedergaard, University of Rochester Medical Center Decisions are customarily a result of the Reviewing Editor and the peer reviewers coming together and discussing their recommendations until a consensus is reached. When revisions are invited, a fact-based synthesis statement explaining their decision and outlining what is needed to prepare a revision will be listed below. The following reviewer(s) agreed to reveal their identity: Edward Hall, Dardiotis Efthimios.

The two reviewers had very different opinion about your study. You will have to carefully address reviewer #1 and also reviewer #2 comments for the article accepted for publication.

Reviewer #1

This is an overly ambitious study that seeks to compare the oxidative damage from a variety of neurological disease-relevant reactive oxygen species (peroxynitrite), free radicals (superoxide, nitric oxide) or the neurotoxic lipid peroxidation-derived carbonyl compound 4-HNE in hippocampal neurons isolated from two rats strains (Dark Agouti,DA, and Piebold Viral Glaxo (PVG)that have difference expression levels of the mitochondrial antioxidant enzyme MnSOD. The DA neurons have a higher expression level of MnSOD than the PVG neurons and as a result are more resistant to protein nitration and 4-HNE modification and less damage as measured by LDH release. The main conclusion of this study is that different phenotypic levels of antioxidant enzymes will protect against oxidative damage. First of all that fact is already well known from transgenic antioxidant enzyme over-expressing or antioxidant enzyme knockout animals.

Secondly, while the comparative peroxynitrite and 4-HNE effects on the DA vs. the PVG neurons has merit, the comparison of the effects of superoxide and nitric oxide radicals in isolation reveals the fact that the investigators do not have an accurate knowledge of the complex chemistry of superoxide and nitric oxide which for each is a mix oxidant and antioxidant effects when examined by themselves.

Thirdly, while MnSOD, is an important antioxidant enzyme that serves to convert superoxide leaking from mitochondrial complex I under physiological conditions, in an injury or ischemic setting, the increase in calcium influx causes the activation of mitochondrial nitric oxide synthase which generates nitric oxide which reacts with superoxide with a rate constant much faster than the rate constant for the reaction of MnSOD with superoxide radical. Thus, relevant to real world pathophysiological situations, the main ROS species to be concerned with in regards to both protein nitration and lipid peroxidation is peroxynitrite. Therefore, the experiments comparing damage by application of superoxide or nitric oxide radicals in isolation are really meaningless.

Reviewer #2

The authors aimed to investigate whether antioxidative response differs between phenotypes and whether it affects the severity of the secondary injury, by comparing the antioxidative response in inbred rat strains Dark Agouti to Piebald Viral Glaxo. They concluded that phenotypic antioxidative regulation may affect the secondary inflammation.

The manuscript is generally well-witten.

My only concern is that the title of the manuscript does not correspond to the concept of the experiment as no genetic polymorphisms were studied. Instead genetic background would be more appropriate.

## Author Response

Dear Editor and Reviewers,

Thank you for your valuable remarks regarding our manuscript. We here provide a detailed response, in addition to the revised manuscript.

Reviewer #1

1

Reviewer #1 correctly points out that earlier studies of knock-out animals have established that manipulating specific enzymes may affect the redox balance (1). The current study aimed to compare rat strains with defined genetic backgrounds with known susceptibility to CNS inflammation, based on earlier studies suggesting links between CNS inflammation-susceptibility and oxidative stress. The DA and PVG strains are not genetically manipulated to under- or overexpress specific enzymes. We provide an alternative approach to test genetic control of oxidative insults. The study presents novel findings of genetic control of redox processes, in inbred and not engineered genomes. Knock-out models may be contradictive. For example, conditional knock-out of MnSOD in postnatal neurons in mice does not result in an increase in oxidative damage (2). It is a relevant point that was not sufficiently clarified in the discussion, and is now revised in the manuscript.

In addition, we have added references and discussion to frame the study within the perspective of individual heterogeneity in patients suffering from head injury; a condition that is still without pharmacological neuroprotection is spite of extensive trials of pre-clinically validated drugs. In this context, we have introduced the idea of a ‘systems-response’ of the inflammatory systems as a model to study the integrated response of an individual to a potentially damaging stimulus.

2, 3

Reviewer #1 points out that superoxide and nitric oxide have both oxidant and antioxidant properties and that adding only one compound does not resemble the real world. We agree that the system is artificial, as is the cell culture environment with raised CO2 levels, cell medium nutrition and the omission of supporting cells. Cell culture studies should therefore not claim to be automatically transferable to the real world, but they complement animal and human studies to test hypotheses that may not be tested in vivo. We set out to test the hypothesis that neurons have different susceptibility to oxidative stress, which would not be possible to test in vivo with the methodology available of today. Our primary hypothesis was tested by SIN-1, a compound that releases nitric oxide and superoxide simultaneously to create peroxynitrite. We then aimed to further explore the effects of oxidants in DA and PVG neurons, using high concentrations of compounds also known to create peroxynitrite; nitric oxide and superoxide, knowing that the intracellular milieu in the cell culture was not void of other oxidants. The rationale was not to test the individual oxidative properties of superoxide and nitric oxide in neurons specifically, and superimpose the result to an in vivo environment, but to test the concept of different regulation between DA and PVG, using a multitude of oxidants, in a reductionist environment. We aimed to describe how DA and PVG neurons may vary in the response to oxidative stimuli, while the specific oxidative pathophysiology of the neurons was beyond the scope of this report.

Reviewer #2

We have changed the title according to the suggestion of reviewer #2.

References

1. Holley AK, Bakthavatchalu V, Velez-Roman JM, St. Clair DK: Manganese Superoxide Dismutase: Guardian of the Powerhouse. In Int J Mol Sci, 2011, p. 7114-7162

2. Misawa H, Nakata K, Matsuura J, Moriwaki Y, Kawashima K, Shimizu T, Shirasawa T, Takahashi R: Conditional knockout of Mn superoxide dismutase in postnatal motor neurons reveals resistance to mitochondrial generated superoxide radicals. Neurobiol Dis 2006;23:169-177
